# Isolated pneumomediastinum in severe category COVID-19 patients admitted in ICU: a case series

**DOI:** 10.1186/s42077-022-00235-0

**Published:** 2022-04-01

**Authors:** Nazia Nazir, Anupriya Saxena

**Affiliations:** Department of Anaesthesiology and Critical Care, Government Institute of Medical Sciences, 15/6; Block F; Gautam Buddha University Campus, Greater Noida, Uttar Pradesh India

**Keywords:** COVID, Cough, Non-invasive ventilation, Pneumomediastinum

## Abstract

**Background:**

Pneumomediastinum is a rare complication associated with acute respiratory distress syndrome secondary to other viral pneumonias. However, it has been on a rise in COVID-19 patients with severe disease.

**Case presentation:**

We present three cases to highlight that isolated pneumomediastinum can complicate the course of illness in patients on non-invasive ventilation. In our case series, two COVID-19 diagnosed cases with no previous history of intubation developed pneumomediastinum. They were managed conservatively with a successful outcome. The third patient developed PM with subcutaneous emphysema post-intubation, although managed conservatively succumbed to the disease.

**Conclusions:**

Clinicians need to be alert to the development of such a complication in the event of sudden onset dyspnoea with chest pain. Conservative management, with low pressure settings on the ventilator results in gradual improvement of patient.

## Background

Pneumomediastinum (PM), while not very common in other viral pneumonias, is being increasingly reported as a complication of acute respiratory distress syndrome (ARDS) due to COVID-19 (Volpi et al. 2020).

## Case presentation

We here describe three cases of laboratory and radiology confirmed COVID-19 who were diagnosed with isolated PM over the course of intensive care unit (ICU) stay.

### Case 1

**Fig. 1:**
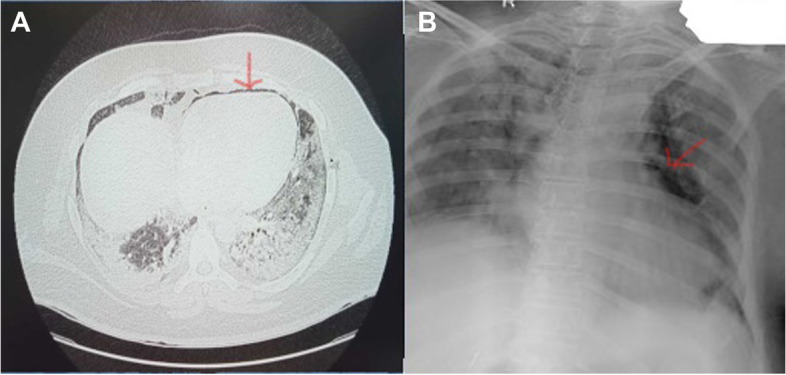
**A** HRCT on admission. **B** CXR showing pneumomediastinum (red arrows)

A 53-year-old female, weighing 104 kg (BMI 40 kg/m^2^) presented with fever and cough for 5 days, mild shortness of breath for 3 days with a history of hypertension for the last 3 years. Reverse transcriptase polymerase chain reaction (RT-PCR) was positive for COVID-19 with a cycle threshold (ct) value for N-gene 18/30. On admission, she maintained a saturation (SpO_2_) of 92% on room air. She was started on O_2_ support @5 l/min with a Hudson mask, after which her SpO_2_ improved to 95%. Initial X-ray chest (CXR) revealed haziness and alveolar opacities both lungs in lower zones. High-resolution CT scan (HRCT) showed patchy areas of ground-glass attenuation with septal thickening scattered in bilateral lungs, more so in subpleural and lower lobes (Fig. 1A). The CT severity score was 18/25. Her laboratory reports revealed, white cell count (WBC) 15 thous cells/cubic mm, platelets 2.28 lakh cells/cubic mm, procalcitonin (PCT) 0.19 ng/mL, C-reactive protein (CRP) 71 mg/L, D-dimer 0.24 mg/l FEU, lactate dehydrogenase (LDH) 846 U/L, and ferritin > 1226 ng/mL. She was initiated on a course of injection (inj.) remedesivir, convalescent plasma therapy (CPT), inj. enoxaparin, and inj. imipenem, along with inj. methylprednisolone. After 2 days, she developed sudden respiratory distress with a drop in SpO_2_. The patient was started on non-invasive ventilation (NIV) with pressure support (PS) 8 mmHg and positive end-expiratory pressure (PEEP) 6 mmHg. She was maintained on intermittent NIV and high flow nasal oxygen therapy (HFNC) in a 4:1 ratio. On day 13, she complained of chest pain for which an electrocardiogram and CXR were done revealing isolated PM (Fig. 1B). The patient did not require endotracheal intubation and was managed on NIV with low-pressure settings. Cardiothoracic surgery opinion was sought who advised for conservative management as the patient was hemodynamically stable. At the time of writing this article, she was in stable condition and de-escalated to HFNC 25 L/min, and follow-up CXR also shows resolution of lung shadows and absorption of PM.

### Case 2

**Fig. 2:**
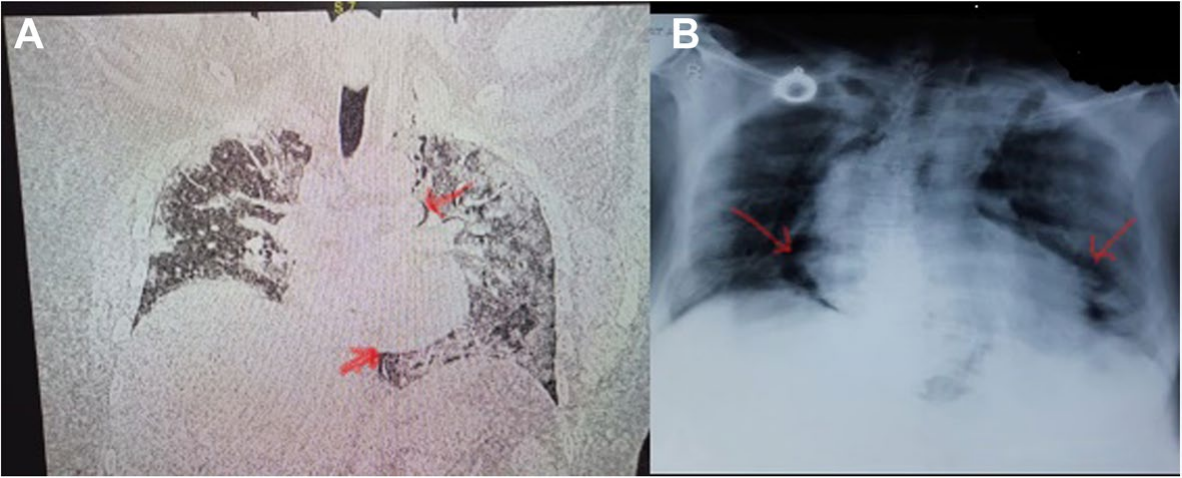
**A** HRCT on admission. **B** CXR showing pneumomediastinum (red arrows)

A 45-year-old male, weight 70 kg (BMI 23 kg/m^2^) and with no comorbidity, presented to the COVID outpatient department with cough and shortness of breath for 5 days and SpO_2_ of 90% on 10 L O_2_ support. RT-PCR was positive for COVID-19 with a ct value for N-gene 13/30. He was admitted to the ICU and put on a non-rebreathing mask with O_2_ @ 12 L/min. CXR on admission revealed haziness and alveolar opacities in both lungs and HRCT was reported to have bilateral ground-glass opacities with few subpleural bands noted in the right lower lobe (Fig. 2A) (CT severity score 15/25). His laboratory workup was as follows: WBC 10 thous cells/cubic mm, platelets 1.2 lakh cells/cubic mm, PCT 0.11 ng/mL, CRP 140 mg/L, LDH 1320 U/L. He received treatment with inj. remedesivir, inj tazobactam piperacillin,inj enoxaparin, and inj. methylprednisolone. Five days later, he required O_2_ @ 60 L/min HFNC to maintain oxygen saturation ≥ 90%. On the next day, he was switched to NIV with PS 11 mmHg, PEEP 6 mmHg in view of increased work of breathing. On day 12, the patient developed increased respiratory distress with central chest pain. ECG revealed sinus tachycardia and CXR an isolated PM (Fig. 2B). The PM was managed conservatively and NIV with low pressure setting was continued. Serial imaging by CXR confirmed the resolution of the PM. He continued to improve and was weaned off oxygen support gradually. The patient was eventually discharged to the ward on day 28.

### Case 3

**Fig. 3:**
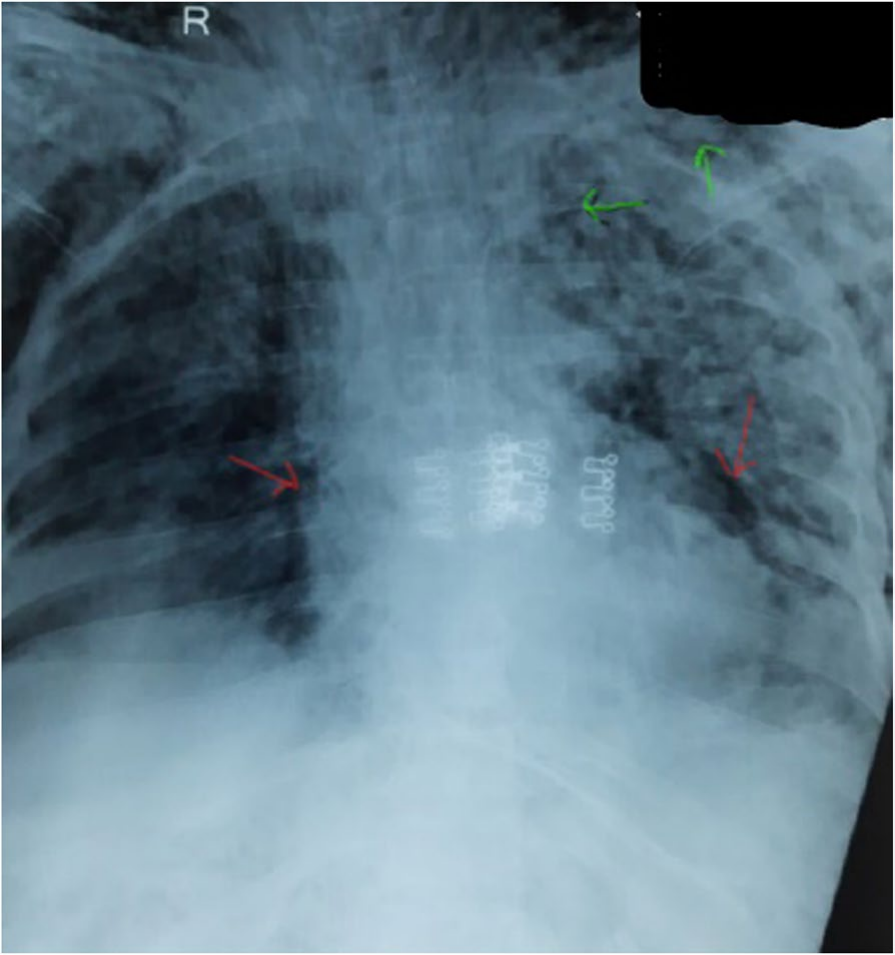
CXR showing pneumomediastinum (red arrows) with subcutaneous emphysema (green arrows)

A 60-year-old female, weighing 96 kg (BMI-35 kg/m^2^) with no comorbidity was presented to hospital with shortness of breath, cough, and chest pain for 10 days with a SpO_2_ of 91% on 12 L O2 support. She was referred from a private hospital. She tested positive for COVID 19 with a ct-value for N-gene 25/30. On admission, CXR revealed haziness and alveolar opacities in both lungs, suggestive of ARDS. HRCT was not available. Laboratory workup was WBC 18 thous cells/cubic mm, platelets 1.06 lakh cells/cubic mm, CRP protein 114 mg/L, LDH 1110 U/L. Her treatment consisted of inj. remedesivir, CPT, inj. tazobactam piperacillin, inj. enoxaparin, and inj. methylprednisolone. Her oxygen therapy rapidly escalated as the patient did not maintain saturation and was intubated on day 14 of illness. CXR on day 14 revealed patchy opacities bilateral mid and lower zones with isolated PM and subcutaneous emphysema in the anterior chest wall (Fig. 3). In view of hemodynamic stability, conservative management for the PM was advised. On serial imaging, the PM did not show an increase; however, over the course of illness, the patient developed ventilator-associated pneumonia and septic shock. She suffered from cardiac arrest and passed away on day 19 of illness.

## Discussion

Pneumomediastinum is defined as the presence of interstitial air in the mediastinum. It can be either spontaneous, secondary to any intervention, trauma or organ injury, mechanical ventilation, or any intrathoracic infection (Mohan and Tauseen 2020).

Recently there have been reports of increased incidence of PM, pneumothorax and subcutaneous emphysema in COVID-19 patients (Mohan and Tauseen 2020; Agrawal et al. 2021; Kangas-Dick et al. 2021). Our case series focuses on the development of isolated PM, in the two COVID-19 diagnosed cases with no previous history of intubation managed conservatively with a successful outcome. The third patient developed PM with subcutaneous emphysema post-intubation, although managed conservatively succumbed to the disease.

In our patients, PM or subcutaneous emphysema could be attributed as a sequela of COVID-19 with prolonged non-invasive ventilation. All of our patients had common symptoms of dyspnoea with cough. Increased alveolar pressure and diffuse alveolar injury are pathognomonic of COVID-19 pneumonia. This makes the alveoli more prone to rupture especially in patients with pronounced cough (Mohan and Tauseen 2020). The resultant alveolar rupture causes PM through Macklin’s phenomenon (Murayama and Gibo 2014). Although spontaneous pneumothorax and PM is a well-documented complication of COVID-19 pneumonia (Mohan and Tauseen 2020; Agrawal et al. 2021), all of our patients were on NIV prior to the development of PM. Hence, PM should be considered as an alternative cause of acute deterioration in COVID-19 pneumonia patients on non-invasive positive pressure ventilation therapy.

It is of note that none of our patients gave any history of smoking or any respiratory ailment in the past. A positive correlation between pneumothorax and steroid use in ARDS patients has been postulated by some authors (Wali et al. 2020). However, a similar relationship between PM and steroid use cannot be established due to confounders such as age, body habitus, and duration of ventilation (Baek et al. 2021). Tracheal manipulation and procedural airway injury have also been implicated as possible causes of PM in COVID-19 (Wali et al. 2020). However, two of our patients developed PM in absence of any tracheal manipulation.

Although HRCT remains the definitive diagnostic tool for PM (Kouritas et al. 2015), it requires transferring patients to and from the radiology suite. However, in COVID-19 patients, such transfer creates logistic issues and serves as a source of potential breaches in COVID protocols. Kouritas et al. have clearly stated that “the diagnosis of PM can usually be established with a plain anterior chest film, showing lucent streaks, bubbles of air outlining mediastinal structures, and visible mediastinal pleura. This can yield a diagnosis in almost 90% of reported series (Kouritas et al. 2015).

## Conclusions

By presenting these 3 cases, we emphasize that isolated pneumomediastinum not associated with pneumothorax can be managed conservatively and may not necessarily result in a poor outcome. However, monitoring of patients for clinical deterioration and serial CXR for diagnosing the development of associated pneumothorax is of vital importance.

## Data Availability

All data generated or analyzed during this study are included in the published article [and its supplementary information files].

## Abbreviations

PM: Pneumomediastinum
ARDS: Acute respiratory distress syndrome
COVID: Coronavirus disease
ICU: Intensive care unit
BMI: Body mass index
RT-PCR: Reverse transcriptase polymerase chain reaction
ct: Cycle threshold
SpO2: Saturation of oxygen in blood
CXR: Chest X-ray
HRCT: High-resolution chest tomography
WBC: White blood cell
PCT: Procalcitonin
CRP: C-reactive protein
LDH: Lactate dehydrogenase
Inj: Injection
CPT: Convalescent plasma therapy
NIV: Non-invasive ventilation
PEEP: Positive end expiratory pressure
HFNC: High floe nasal cannula
ECG: Electrocardiogram
